# A Practical Framework for Environmental Antibiotic Resistance Monitoring in Freshwater Ecosystems

**DOI:** 10.3390/antibiotics14080840

**Published:** 2025-08-19

**Authors:** Irene Beltrán de Heredia, Itziar Alkorta, Carlos Garbisu, Estilita Ruiz-Romera

**Affiliations:** 1 Department of Chemical and Environmental Engineering, University of the Basque Country (UPV/EHU), Plaza Ingeniero Torres Quevedo 1, 48013 Bilbao, Spain; estilita.ruiz@ehu.eus; 2 Department of Biochemistry and Molecular Biology, University of the Basque Country (UPV/EHU), P.O. Box 644, 48080 Bilbao, Spain; itzi.alkorta@ehu.eus; 3 Department of Conservation of Natural Resources, NEIKER–Basque Institute for Agricultural Research and Development, Basque Research and Technology Alliance (BRTA), Bizkaia Science and Technology Park, P812, 48160 Derio, Spain; cgarbisu@neiker.eus

**Keywords:** antimicrobial resistance, aquatic environments, monitoring, one health, surface water, surveillance, wastewater

## Abstract

Antibiotic resistance (AR) and contamination are critical public and environmental health issues. In the last years, the environmental component of AR has acquired much interest due to its potential links with the human resistome. In particular, freshwater ecosystems are considered strategic sites for environmental AR surveillance, since they can act as both reservoirs and transmission routes for antibiotic-resistant bacteria and antibiotic resistance genes. Many studies are needed to deepen our understanding of AR evolution and dynamics in freshwater ecosystems and, specifically, on the existence of links between environmental and human AR. This calls for the design of robust and adaptive AR surveillance strategies and, concomitantly, the implementation of routine monitoring programs that effectively capture the environmental dimension of AR in freshwater ecosystems. Here, a roadmap for AR monitoring in freshwater ecosystems, framed around four essential questions (how? what? where? when?), is presented to guide researchers and decision-makers in designing and implementing effective environmental AR routine monitoring programs. It was concluded that, due to the complexity, heterogeneity, and dynamic nature of freshwater ecosystems, it seems foreseeable that environmental AR monitoring programs need to be carefully adjusted to the particular casuistry of each freshwater ecosystem, as well as to the specific interests of the corresponding program and the resources available. Still, much research is needed to properly assess and monitor the risks derived from the emergence and dissemination of AR determinants in freshwaters for both ecosystem and human health. By synthesizing current knowledge and methodologies, this review consolidates existing approaches and can serve as a guide for planning AR monitoring programs in freshwater ecosystems.

## 1. Why Environmental AR Monitoring Is Important

Antibiotic resistance (AR) is a global health problem, currently compromising a great number of medical treatments and practices. The e effective control of AR emergence and spread demands coordinated efforts following the “One Health” framework, which emphasises the many links and close interdependence among human, animal, and environmental health [1,2,3]. Understandably, initially and for a long time, research on AR was mainly focused on its clinical relevance, especially in hospital settings. But, in the last decades, the environmental component of AR has been increasingly recognised and studied [4,5].

Particularly, aquatic systems have been found to play a pivotal role in AR, serving simultaneously as reservoirs, mixing zones, and dissemination routes for antibiotic residues, antibiotic-resistant bacteria (ARB), and antibiotic resistance genes (ARGs) [6,7]. Among these, freshwater ecosystems are highly relevant as they serve as the primary source of drinking water, support recreational activities, and sustain agricultural irrigation and freshwater aquaculture. Additionally, freshwater ecosystems are most frequently subjected to contamination from a variety of sources (e.g., agricultural runoff, livestock waste, untreated or partially treated wastewater, industrial discharges, mining operations, and effluents from aquaculture activities), as well as to climate change-derived environmental impacts [8,9], which may amplify AR risks. In consequence, freshwater ecosystems are nowadays recognised as critical sites for environmental AR surveillance.

Despite the current agreement on the need for environmental AR monitoring, many more studies are required to deepen our understanding on AR evolution and dynamics in freshwater ecosystems and, specifically, on the existence of potential links between environmental and human AR. Following an adaptive environmental assessment and management framework, long-term monitoring programs must be designed and evolved iteratively as new information and research questions emerge, emphasizing the criticality of posing at the outset well-defined questions, as well as testable hypotheses and objectives. Thus, environmental AR monitoring programs need to (i) adapt to evolving detection capabilities; (ii) incorporate new AR targets; and (iii) account for spatial and temporal variability. Also, to maximise the accomplishments of surveillance efforts while ensuring an efficient use of resources, before taking samples, it is crucial to define the parameters and analytical techniques of interest in order to properly outline the sampling strategy. In any case, the design of robust environmental AR routine monitoring programs still remains a substantial challenge [10].

**Figure 1:**
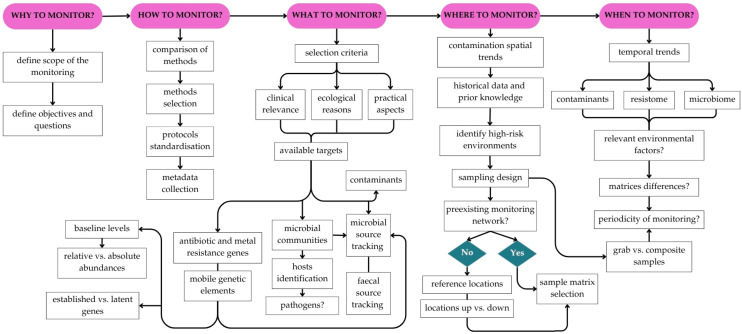
Roadmap for routine antibiotic resistance (AR) monitoring in freshwater ecosystems (created with https://www.canva.com (accessed on 16 July 2025)).

In consequence, this review aims to provide clear guidance for environmental antibiotic resistance monitoring in freshwater ecosystems. To this end, a roadmap is presented here to support the practical implementation of routine AR monitoring programs in those systems (Figure 1). Once the importance of AR monitoring (why?) is established, the roadmap is organised around four essential questions—how? what? where? when?—which serve as conceptual pillars for structuring the abovementioned monitoring programs. By synthesizing current knowledge and methodologies, we aim to (i) integrate and condense a wide range of aspects into a coherent and accessible format; (ii) facilitate its adoption by regulatory authorities and decision-makers; and, ultimately, (iii) strengthen environmental and public health protection efforts.

## 2. How to Monitor?

### 2.1. Methodologies for AR Monitoring

In the past, our understanding of AR mainly relied on data provided by culture-dependent methods [11]. While informative, traditional culturing and susceptibility testing are often labour-intensive and time-consuming [12]. Additionally, culture-dependent methods have important limitations for most environmental bacteria, as the majority of bacterial species present in natural ecosystems are not cultivable in laboratory settings using current techniques [13,14,15]. On the other hand, the cultivation of bacterial strains in the presence of antibiotics may distort AR results by triggering or amplifying ARGs present at very low abundances in the natural environment prior to strain cultivation.

The development of molecular tools and sequencing technologies has revolutionised the microbial ecology field and, in particular, environmental AR monitoring [16]. Most current environmental AR studies rely on PCR technology to detect a selected group of ARGs and mobile genetic element-linked genes (MGE-linked genes) involved in AR dissemination [17,18]. PCR and real-time quantitative PCR (qPCR) are among the most widely used methods for detecting ARGs in environmental samples, mainly due to their high sensitivity, analytical speed, and relatively low cost [19]. Nonetheless, traditional PCR-based approaches, while extremely useful, yield limited and biased information on a small subset of selected genes [20,21]. Multiplex PCR and high-throughput PCR arrays can be extended to hundreds of ARGs in a single run, but require stringent controls and very careful primer design [15,22].

Next-generation sequencing (NGS) technologies, including a variety of powerful and promising approaches [23], are based on the extraction and sequencing of nucleic acids from environmental samples, followed by analysis of the resulting reads using reference databases. In targeted sequencing (e.g., amplicon-based metabarcoding), PCR-amplified barcoding regions (e.g., 16S rRNA for prokaryotes, 18S rRNA for eukaryotes, ITS for fungi) are often used to determine microbial community structure and composition [24]. Shotgun metagenomics enables the simultaneous analysis of microbial community structure and function without the preselection of specific genes as targets [16,18]. This methodology can also reveal the genetic context of the identified genes, a crucial fact for a better understanding of the risks and mechanisms involved in ARGs spread between cells and across environments [25], and, in particular, the potential associations between ARGs in environmental bacteria and human pathogens [26]. Importantly, metagenomics techniques have emerged as powerful and promising tools for effectively monitoring freshwater bodies, offering rapid and high-resolution profiling, and enabling scalable and comparative analyses across multiple sites and samples. Finally, whole-genome sequencing of environmental bacteria is also possible, but normally limited to cultivable strains [24].

For a much more detailed information on these and other molecular and sequencing methods for studying microbial communities and genes from environmental samples and, specifically, for the analysis of AR, readers are referred to outstanding reviews [16,21,27,28,29,30,31].

### 2.2. Criteria for the Selection of Methods

A key aspect of routine monitoring surveillance is protocol harmonisation. Given the variety of methods for environmental AR monitoring, protocol harmonisation and standardisation are critical to ensure comparability and interoperability among studies. However, it is very difficult to reach a consensus on which methods provide the most valuable information, since each method has advantages and disadvantages, and the trade-offs between them are often irresolute if not irresolvable (Table 1).

If we had to start somewhere, perhaps one of the initial points to consider would be the economic affordability of the environmental AR monitoring method. While the scientific value provided by each method should be, theoretically speaking, the primary criterion, cost-effectiveness and economic feasibility are, in practice, equally important since they provide accessibility for scientific groups, thereby facilitating widespread adoption. Another most relevant aspect is protocol applicability, since the selected methods should be easy to use and, preferably, deliver rapid results. Labour-intensive protocols requiring high expertise and time-consuming sample preparations are unlikely suitable for large-scale monitoring programs. Building upon existing surveillance systems that already include microbiological analyses can reduce environmental AR monitoring costs while improving implementation efficiency [10].

Technological advances in DNA sequencing have drastically reduced costs during the past decade, making it economically affordable for many research groups [24]. Yet, data analysis remains a bottleneck owing to the (i) vast volumes of data generated (ranging from giga- to terabases); (ii) the substantial amounts of computational resources required; and (iii) the bioinformatics expertise needed to fully leverage the potential of metagenomes. Nevertheless, recent advances in bioinformatics have led to the development of new specialised tools and simplified downstream analyses [32,33].

Ultimately, the suitability of each method depends, among other aspects, on the specific questions to be answered, the monitoring goals, the available information, the accepted knowledge and values, and the intended actions to be taken according to the obtained results [19,34]. When the objective is limited to only detect a few known ARGs expected to confer a specific AR phenotype, e.g., to detect specific high-risk ARGs, PCR-based technologies may be an appropriate choice due to their sensitivity and rapid provision of data on, for instance, potential deviations from gene abundance background levels. PCR-based technologies are also commonly used to determine the abundance of MGE-linked genes, in order to gain a general idea of the dissemination potential of ARGs. Conversely, if the aim of the study is to identify potential emerging AR threats and delve into their genetic context, shotgun metagenomics might be the most suitable option.

In any event, the first step towards standardisation is to assess and compare the methods currently in use to monitor environmental AR [10,27]. Such evaluations are critical to understand to which extent the results obtained using one approach align with those obtained through alternative approaches. However, such comparisons have so far been conducted only in a limited number of settings where AR was being monitored (Table 2; see also Table S1 in Supplementary Material, providing methodological details). Despite this, early investigations on AR are paving the way for broader evaluations. The new efforts will clarify which measurements are mutually informative and which offer the most comprehensive overview of the AR landscape in a given environment.

**Table 2:** List of studies comparing methodologies for AR analysis in aquatic systems or wastewater.

Study System *	Methods Compared **	Main Findings **	References
WASTEWATER Influent samples collected daily over 18 consecutive days and used to create a composite sample	qPCR SM-Seq RNA-seq 16S rRNA-seq	qPCR adequately identified, relatively quantified and validated ARGs identified by SM-Seq and RNA-seq RNA-seq detected only 32% of ARGs identified by SM-Seq All ARGs detected by RNA-seq were also detected via SM-Seq albeit at varying relative proportions SM-Seq with MEGARes gave better resolution than CARD SM-Seq and 16S rRNA-seq were equally effective at phylum/class level, but SM-Seq revealed greater species richness and higher abundance	[35]
WASTEWATER Post screen influent, treated effluent (drop chamber after final clarifiers), tertiary maturation pond effluent, (final pond prior to discharge), pond base sediment	qPCR SM-Seq	qPCR was more sensitive, particularly in diluted samples with low ARG concentrations SM-Seq was more specific with less off-target risk in concentrated samples SM-Seq revealed multiple gene subtypes that qPCR could not distinguish Both methods are suitable for profiling wastewater resistomes depending on the sample and research needs	[36]
RIVER WATER River water samples upstream and downstream of 3 reclamation plants, swimming/kayak sites, and beaches near coastal pour	CBM qPCR SM-Seq	qPCR, SM-Seq, and culture-based methods showed similar ARG abundance trends in the watershed. qPCR and SM-Seq detected dilution gradients across land uses. SM-Seq better detected and quantified stepwise ARG changes and covered more ARG classes qPCR was more sensitive in marine samples than metagenomics Culture-based and SM-Seq detected high AR areas, qPCR did not qPCR and SM-Seq effectively reveal ARG trends related to land use and contamination, with added value from culture viability methods	[37]
WASTEWATER Four wastewater samples from hospital, industrial, urban, and rural areas	HT-qPCR SM-Seq	Strong correlation in ARG relative abundances between the two methods for most antibiotic classes qPCR more prone to false negatives from mutated primer target sites SM-Seq missed ARGs with incomplete or low coverage due to pipeline settings Combining both methods improves robustness of ARG surveillance	[38]
WASTEWATER Wastewater influent from 47 WWTPs	HT-qPCR SM-Seq	SM-Seq offered broader resistome coverage and host context for risk assessment HT qPCR was more sensitive, quantifying targeted genes including low-abundance clinically relevant genes Both methods captured spatio-temporal resistome patterns and distinguished hospital vs. WWTP profiles Both approaches showed links between resistome changes and environmental factors, but interpreted drivers differently	[39]
MULTIPLE WATER SOURCES Water samples collected from groundwater, surface water, drinking water treatment plants before pre-treatment, and tanker filling stations	qPCR HT-qPCR	HT-qPCR results were in agreement with those from standard qPCR Both methods showed comparable performances as well as successful detection of MST markers in faecal source Successful detection of MST markers in faecal and water samples shows their potential for identifying faecal contamination via HT-qPCR	[40,41,42,43,44]
RIVER WATER AND WASTEWATER Water and wastewater samples from hospital effluent, two WWTP treatment stages, and river receiving discharge point	qPCR SM-Seq	Both methods distinguished resistome profiles and detected gradient stepwise mixtures, but qPCR was more sensitive for some ARGs qPCR showed higher accuracy in predicted and observed ARG quantification SM-Seq provided a markedly higher ARG coverage despite lower sensitivity	[45]
MULTIPLE WATER SOURCES Wastewater, recycled water, and surface water samples collected over six months from six utilities, in five US states	CBM qPCR	Both methods consistently reflected the same trends showing highest AMR levels in raw wastewater and lowest in recycled water effluents qPCR measurements were significantly correlated with culture-based measurements across all sample types, though correlation was moderate qPCR detection of *sul1* yielded the widest dynamic range of measurement as an AR indicator, while *intI1* was the most frequently detected target	[46]
RIVER WATER Urban watershed before and after a rainfall event	CBM qPCR SM-Seq	All methods detected an increase in faecal contamination from multiple sources, FIB, enteric microbes, and ARGs after a storm event Both CBM and qPCR consistently detected *E. coli* and enterococci. SM-Seq reflected similar trends in faecal indicator bacteria (FIB) relative abundance but with comparatively lower counts, supporting its complementary rather than standalone use SM-Seq provided finer taxonomic resolution of faecal-associated microbes, while qPCR markers closely correlated with viral SM-Seq indicators Rapid qPCR for enterococci and *E. coli* offers a shorter sample processing time compared to FIB culturing, while SM-Seq expands coverage beyond culture and qPCR targets, enhancing water quality insights SM-Seq limited to relative abundance shifts and does not provide absolute quantification, viability, or infectious potential of detected microbes	[47]
WASTEWATER Grab samples were collected at each stage of treatment	qPCR SM-Seq	Spearman correlation analysis showed significant agreement between qPCR ARG abundances and SM-Seq calculated absolute abundance Significant correlations were observed between both methods for the quantification of *ermB*, *sul1*, and aggregated *bla_TEM_* genes *vanA* was only detected by SM-Seq, preventing correlation to qPCR data and suggesting caution due to potential biases in sequencing	[48]
FRESHWATER RESERVOIR Subtropical stratified freshwater reservoir	HT-qPCR SM-Seq	SM-Seq detected more ARG subtypes and much higher abundances of bacitracin ARGs than HT-qPCR Both methods revealed similar ARG spatio-temporal patterns, ARGs-bacterial taxa co-occurrences, and environmental effects on ARG profiles HT-qPCR has advantages such as time-saving, absolute quantification, and low bioinformatics requirements, but is limited by PCR amplification and primer bias and lower ARG subtype coverage compared to SM-Seq HT-qPCR is suitable for routine aquatic monitoring, while SM-Seq is an ideal tool for more comprehensive survey of environmental ARG subtypes	[49]

* Samples indicated for each system refer only to water samples from different sources. ** Only the most relevant results regarding method comparison are highlighted. CBM: culture-based methods; SM-Seq: shotgun metagenomic sequencing; RNA-seq: RNA sequencing; 16S rRNA-seq: 16S rRNA gene sequencing.

### 2.3. Challenges in Resistome Profiling via Metagenomics

While NGS technologies have undoubtedly revolutionised the study of microbial communities and, in particular, bacterial AR, it is important to acknowledge the inherent limitations of metagenomic sequencing. Similarly, it is crucial to determine whether the general guidelines applied to sequencing practices in other fields are appropriate for the analysis of the environmental resistome. Key challenges that need to be addressed include the following areas:

1. The detected ARG diversity does not capture the whole diversity: In many environmental samples, ARG relative abundance is often much lower than that of other functional genes [15], requiring deep sequencing to capture the whole resistome diversity [50]. Without adequate sequencing depth, crucial ARGs may be underrepresented, leading to incomplete or misleading conclusions. Likewise, expanding reference databases to include both clinical and environmental genome data, along with the integration of predictive models, is essential. Current databases are biased toward model organisms, pathogens, and easily cultivable bacteria [51]. Also, in the context of freshwater ecosystems, obtaining a sufficient amount of DNA from a given sample, in order to allow a full characterization of the microbiome and resistome, can be a demanding task, due to the common relatively low bacterial densities per unit of water, which often necessitate large sample volumes (e.g., >1 L) to perform metagenomic analyses effectively [27].
2. Absence does not mean susceptible: Failure to detect an ARG does not necessarily imply that such gene is not there or that the host bacterium is susceptible to the antibiotic in question. In addition to detection limits (sensitivity) and difficulties, AR may (i) involve mechanisms beyond those identifiable through gene sequencing data; and (ii) not depend on the presence of specific ARGs. Furthermore, certain bacteria harbour silent ARGs, whose presence is not associated with a corresponding resistant phenotype, but that, under suitable conditions, can revert their expression patterns and result in AR [52].
3. Presence does not imply functionality: DNA-based methods, when used alone, do not reveal whether putative target genes are functional or actively expressed in the environment [53]. While genomic content indicates the functional potential of a microbial community, it fails to directly measure actual functional activity. This limitation can be addressed by integrating complementary approaches, such as metatranscriptomics, metaproteomics, and metabolomics, which enable the analysis of gene expression, protein production, and metabolic activity, respectively [54]. Likewise, DNA can persist in the environment for a relatively long time after cell death, but molecular techniques cannot differentiate between living and dead organisms, and then sequencing results may not accurately represent the active microbial populations [51]. However, detecting the presence of ARGs is always important, as extracellular DNA can be taken up via transformation and then expressed by the host bacteria [5,55].

Overcoming these limitations requires both innovative approaches and the integration of complementary methods, including traditional cultivation and molecular sequencing methods. Continued research and refinement of sequencing techniques are needed, but also the use of theoretical and practical approaches that exploit both cutting-edge and well-established methods.

### 2.4. Standardisation of Protocols

A carefully designed monitoring program is crucial when studying the environmental resistome and, in consequence, must be meticulously and methodically planned to properly address the research questions of interest. The incorporation of best practices for data generation and processing is also essential to ensure comparability across spatial and temporal scales. Key considerations include (i) sampling strategy, which can include grab (active), composite (time-, flow-, or spatially weighted), and passive sampling methods; (ii) sample types, including water, wastewater, sediments, biofilms, and aquatic organisms, among others; (iii) sampling frequency and biological replication, based on monitoring objectives and the spatio-temporal variability of the system under consideration (lower frequency for stable environments vs. higher frequency for heterogeneous or variable systems); (iv) sample preservation and storage, important to carefully preserve the original state of the samples, which should be handled and processed consistently (i.e., standardized storage time and temperature) within a given monitoring program; (v) sample concentration techniques, which are still being optimised for many environmental matrices; (vi) DNA extraction protocols, which should be selected depending on the specific environmental matrix of interest, be consistent across sample sets intended for comparisons, include quality controls such as negative controls (e.g., field blanks or DNA extraction blanks), positive controls (e.g., a mock community processed as a separate sample) or internal amplification standards (e.g., exogenous whole cells, DNA or RNA added to the sample matrix), and incorporate the quantification of extraction yields (e.g., NanoDrop spectrophotometer or Qubit assay) [20,28,33,56].

**Figure 2:**
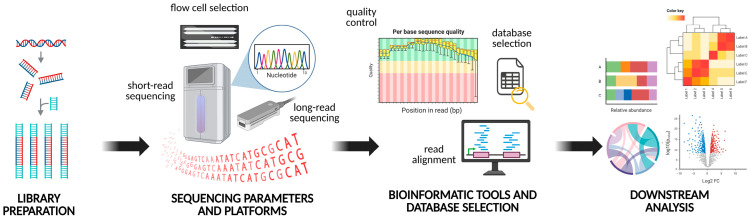
Overview of key decision points to consider in developing a workflow for metagenomics (created with https://www.biorender.com (accessed on 25 July 2025)).

The aforementioned points apply broadly across molecular techniques, but shotgun metagenomics requires additional considerations (Figure 2): (i) consistency in library preparation, often constrained by sample DNA quantity and core facility available options, is vital for comparability across metagenomic studies; and (ii) the sequencing technology/platform and associated parameters must be carefully chosen, balancing sequencing depth and economic cost. The expected microbial diversity within the sample, as well as the need to detect rare sequences and taxa, are critical factors that may require deeper sequencing. Including as many technical replicates per flow cell as possible is recommended to identify potential biases and account for batch effects [33,51,57].

The steps followed for downstream analysis are equally critical and include: (i) bioinformatics tools, which come in a wide variety and offer different analytical parameters that can significantly influence downstream results. To ensure reproducibility and comparability across studies, workflows for analysing metagenomic data should be openly shared and bioinformatics pipelines standardised [57,58,59]; (ii) database selection and curation, where the chosen database should be comprehensive, incorporating clinical and environmental reference genome data to enhance understanding of microbial diversity and resistome detection. Moreover, efforts should address ARG nomenclature discrepancies across the different databases; and (iii) normalisation methods for ARG abundances, for which standardisation is urgently required to ensure comparability and reliability in ARG quantification [33,60]. All these constraints call for standardisation and validation of existing experimental designs, as these factors, among others, may limit the utility of molecular methods for many applications [61].

### 2.5. Importance of Metadata Collection and Raw Data Sharing

Standardized AR monitoring frameworks should ensure that contextual data are consistently collected, recorded, and, subsequently, made accessible. Resistomes and microbiomes are closely linked to the environmental context and field conditions, making it essential to report environmental parameters alongside sample collection and processing protocols, so that data from different studies can be properly compared. Efforts have been made to establish recommended metadata templates [62], through the Minimum Information about a Marker Gene Sequence (MIMARKS) and Minimum Information about any (x) Sequence (MiXS) checklists [63], as well as guidelines for experimental transparency and reporting, including Minimum Information for Publication of Quantitative Real-Time PCR Experiments (MIQE) guidelines [64], Environmental Microbiology Minimum Information (EMMI) guidelines [65], and Strengthening The Organization and Reporting of Microbiome Studies (STORMS) reporting guidelines [66]. However, the specific metadata to be collected ultimately depends on the system under investigation [67].

Especially in the context of NGS technologies, sharing raw data in public repositories, like the NCBI Sequence Read Archive (SRA) [68] or the European Nucleotide Archive (ENA) [69], is fundamental, given that a major advantage of metagenomics is the ability to store and analyse data in retrospect [5,10,33]. Archived metagenomic datasets can be re-analysed as new ARGs and MGE-linked genes are discovered, potentially allowing researchers to trace their emergence over time and examine the genomic context in which they originally appeared. Finally, adhering to findable, accessible, interoperable, and reusable (FAIR) data principles is essential for effective data management and stewardship [70].

## 3. What to Monitor?

### 3.1. Selection of AR Targets

A careful selection of environmental AR monitoring targets becomes particularly important for non-metagenomic methodologies, where targeted approaches are often required. In any case, there is broad consensus that the selection of environmental AR surveillance targets should be guided by the specific purpose of the monitoring campaign or program [71].

In many cases, monitoring ARGs of particular clinical relevance may be advisable, including those (i) associated with MGEs; (ii) conferring resistance to last-resort antibiotics; or (iii) found in ESKAPE pathogens (*Enterococcus faecium*, *Staphylococcus aureus*, *Klebsiella pneumoniae*, *Acinetobacter baumannii*, *Pseudomonas aeruginosa*, and *Enterobacter* spp.) [10,56,72,73,74]. Ecological and environmental reasons may also justify the prioritisation of certain ARGs and MGE-linked genes, including those (i) enriched in human-associated environments; (ii) present in ecosystems closely linked to human activity; (iii) occurring frequently across diverse environments; or (iv) serving as indicators to differentiate between human, animal, and environmental AR sources [10,74]. Finally, practical considerations, such as the availability of well-documented data and standardised detection methods, may support prioritisation [10,56,74]. As mentioned in the previous section, significant progress is still needed in the standardisation of protocols and thus, for the time being, prioritising ARGs that have been extensively studied can be advantageous. Ideally, the selection of AR targets should balance potential information yield with all above considerations.

Environmental AR targets should encompass not only ARGs, but also MGE-linked genes that play a pivotal role in horizontal gene transfer (HGT) and, hence, the dissemination of AR traits among bacterial populations and environments. Mobile genetic elements include bacteriophages, conjugative and mobilisable plasmids, genomic islands, transposable elements, and integrons, each of which is characterised by a distinct molecular mechanism. Bacteriophages mediate gene transfer via viral infection and recombination, whereas plasmids—particularly those equipped with a type IV secretion system (T4SS)—enable conjugative exchange. Genomic islands often confer adaptive traits, such as AR or pathogenicity, and can be mobilised through integrative conjugative elements (ICEs) or integrative mobilisable elements (IMEs). Transposable elements contribute to genome plasticity through site-specific excision and insertion. Integrons can capture and express gene cassettes, playing a critical role in microbial adaptation, and can be embedded within other MGEs. Recent studies underscore the emerging significance of ICEs and IMEs for HGT, complementing and sometimes rivalling the long-established role of conjugative plasmids as major drivers of HGT [75].

Previous studies provide useful clues about particularly informative genes, as compiled in Table 3. From candidate gene lists, selection can then be refined and narrowed down to the most meaningful targets. However, despite existing recommendations, it remains unclear which specific AR targets best predict overall ARG abundances in microbial communities. Diagnostic studies contribute to laying the groundwork for identifying high-priority targets for future environmental AR monitoring efforts, as well as recent advances in high-throughput and non-targeted sequencing methods that enable a more comprehensive and unbiased characterisation across a wide range of environments. Notably, redundancy in the information provided by certain targets has been reported [11,74], suggesting that related genes yielding similar abundance values should not be grouped together in the same monitoring panel, especially under resource constraints. In this context, creating a ranked watchlist of potential emerging ARG threats has been suggested, similar to the approach already used for the surveillance of environmental contaminants. An environment-based ARG watchlist would allow for the adjustment of AR monitoring efforts and could act as an early warning system before these genes reach and become widespread in clinical settings. Such a list could inform the design of diagnostic tests aimed at detecting high-risk ARGs [10].

In addition, it is increasingly recognised that the selection of environmental AR monitoring targets should be extended to include heavy metals and, in particular, metal resistance genes (MRGs), given the well-documented co-selection mechanisms where resistance to metals and antibiotics is simultaneously linked through different pathways: (i) co-resistance, when ARGs and MRGs are physically located on the same genetic element (i.e., a conjugative plasmid or ICE) or coexist within the same bacterial cell; (ii) cross-resistance, when a single resistance gene or mechanism confers resistance to both types of compounds simultaneously; and (iii) co-regulatory resistance, where the expression of resistance systems to metals and antibiotics is controlled by a single regulatory gene or common regulator [76,77]. These co-selection mechanisms occur in the absence of antibiotics [78]. Consequently, monitoring MRGs alongside ARGs provides a more comprehensive understanding of environmental resistance risks and helps identify additional sources and mechanisms contributing to the spread of AR resistance.

**Table 3:** List of studies compiling candidate genes regarded as the most informative for environmental AR monitoring purposes.

Proposed Candidate Genes	Selection Criteria	Study Context	References
*intI1*, *sul1*, *sul2*, *bla_CTX-M_*, *bla_TEM_*, *bla_NDM-1_*, *bla_VIM_*, *bla_KPC_*, *vanA*, *qnrS*, *aac(6′)-Ib-cr*, *mecA*, *ermB*, *ermF*, *tetM*, *aph*	Clinical relevance Prevalence in the environment and frequent in environmental settings subjected to human activities Association with MGEs and/or the potential to be acquired by horizontal gene transfer	Various environmental settings	[72]
*intI1*, *sul1*, *tetW*, *bla_TEM_*, *bla_KPC_*, *vanA*, *mcr-1*	Indicators of ARG mobility and anthropogenic contamination (*intI1*) WHO classification: “Highly Important” (*sul1*, *tetW*), “Critically Important” (*bla_TEM_*) Emerging resistance to last-resort antibiotics (*bla_KPC_*, *vanA*, *mcr-1*)	EPA’s NRSA 2013–2014: Stratified, probabilistic survey of nearly 2000 US river and stream sites	[79]
*intI1*, *sul1*, *tetA*, *bla_CTX-M_*, *vanA*	Clinically relevant (*bla_CTX-M_*, *vanA*) Anthropogenically sensitive (*sul1*, *tetA*) Associated with MGEs and anthropogenic impact (*intI1*) Abundant and human-impact correlated (*sul1*, *tetA*, *intI1*)	Systematic review of 117 peer-reviewed studies qPCR based methodologies Surface water, reclaimed water, and/or wastewater	[56]
*intI1*, *sul1*, *tetA* or *tetG*, *vanA*, *bla_CTX-M_*, *bla_TEM_*, *qnrS*, *sul3*, *tetH*, *aadA2*, *floR*, *ereA*, *mexF*	Clinically relevant (*sul1*, *bla_TEM_*, *bla_CTX-M_*, *qnrS*) Anthropogenic markers or enriched in contaminated environments (*intI1*, *sul1*, *qnrS*) Good predictors of total resistome (*bla_TEM_*) Rarely included in qPCR studies but often abundant (*sul3*, *vanA*, *tetH*, *aadA2*, *floR*, *ereA*, *mexF*)	Review of 150 scientific papers qPCR data on ARGs 12 sample types, across 30 countries, from 2001 to 2020	[11]
*intI1*, *sul1*, *ermB*, *oqxA*, *mexE* (from a total of 56 indicator ARGs grouped into four correlated modules)	Clinical relevance Geographic ubiquity, environmental relevance and abundance in wastewater Mobility and association with MGEs Availability of quantification methods	Network analysis of ARG annotations from metagenomic data 191 wastewater and receiving water samples from 64 countries	[74]

### 3.2. Established vs. Latent Genes

When selecting environmental AR monitoring targets, another important consideration is the distinction between established and latent ARGs, as defined by Inda-Díaz et al. (2023) [80]. While established ARGs are already widespread, well-characterised, and typically included in existing reference databases, latent ARGs are poorly studied or entirely uncharacterised. The former are often linked to clinical resistance and pose known threats to human and animal health, whereas the latter have not yet been observed in human pathogenic bacteria. The acquisition of established ARGs from environmental bacteria would probably only marginally contribute to their proliferation. In contrast, the acquisition of latent genes may represent unknown potentially emerging threats [71,81]. While it remains unclear where and under what circumstances novel resistance traits emerge in clinically relevant bacteria, incorporating both ARG categories (established and latent genes) into surveillance frameworks is crucial for tracking the current AR landscape while simultaneously anticipating future risks [19,71]. Furthermore, the identification of novel relevant AR targets may inform and guide future drug developments by revealing emerging AR mechanisms [5].

Strategies to identify upcoming AR threats involve computational approaches as well as functional metagenomics [5,80]. However, most metagenomic studies rely on reference databases, which are biased towards established genes. Moreover, detecting latent genes often requires substantial sequencing depth, as they are generally assumed to be relatively rare [10]. Rapid advances in sequencing technologies and bioinformatics are steadily improving our understanding of microbial resistomes and enabling the integration of latent genes into curated searchable reference databases [16].

### 3.3. Gene Abundances

Much research is needed to establish baseline AR levels across environments [10,11]. Designing effective surveillance strategies requires comprehensive background data on ARG abundances and prevalence in both pristine and human-impacted settings [33]. Without information on typical abundance ranges, single measurements lack meaningful context, thus complicating associations with potential human or animal risks. Detecting deviations from established background conditions would ascertain locations requiring further investigation, either due to unusually high ARG abundances or as potential point sources of specific AR types. However, reference data remain scarce for most environments and, as noted, no universally agreed-upon gene set for environmental AR monitoring exists.

Another key methodological consideration is the distinction between relative and absolute abundances. Relative measures facilitate comparisons across environments or time points, but may introduce compositional bias, especially when microbial biomass or total DNA content varies significantly across samples. This may lead to misinterpretation of ARG trends unless microbial structure shifts are considered. Consequently, integrating both absolute and relative abundances is increasingly recommended for robust insights into AR dynamics [11,21]. Relative gene copy numbers are best suited for comparing environments with similar biodiversity, whereas absolute counts are preferred when contrasting ecosystems with differing microbial diversity. Moreover, relative measures often indicate the extent to which environmental conditions impose selective pressure for ARGs, whereas absolute numbers are arguably more informative for assessing exposure levels and public health risks [21,56].

Quantitative PCR methodologies enable absolute quantification, while also allowing estimation of relative abundances. For most well-characterised ARGs, typical relative abundance ranges from 10^−5^ to 10^−3^ copies per 16S rRNA gene copy [11]. In contrast, absolute gene abundances cannot be directly determined from metagenomic sequencing alone. Instead, normalisation to a secondary internal metric, such as total sequence reads, 16S rRNA gene reads, or single-copy gene reads, is commonly used as a general approach [21,82]. Hybrid approaches that integrate flow cytometry or qPCR data, as well as the use of internal standard spike-ins, are emerging to convert metagenomic relative abundance estimates into absolute values. Nonetheless, these approaches remain preliminary and require further optimisation and validation [82]. In any case, the wide variety of quantification units underscores the need to establish standardised and meaningful normalisation strategies that enhance quantitative resolution, facilitate communication and knowledge consolidation in this research area, and improve cross-study comparability of metagenomic data [21,60].

### 3.4. Microbial Community Dynamics as Drivers of AR

Another fundamental aspect when designing environmental AR monitoring programs is the identification of potential drivers of AR development and dissemination. Studying microbial community changes in structure and composition is just as important as analysing resistome shifts, since AR trends cannot be fully understood without considering microbial community-level changes.

This is crucial for environmental AR monitoring itself and also for the assessment of the potential effects of contamination sources on AR. Antibiotics and other environmental traditional or emerging contaminants can disrupt microbial community structure and functioning, causing both direct (short-term) and indirect (long-term) effects on microbial communities [83]. Many studies have shown that contaminants can cause a reduction in microbial diversity, with disappearance or inhibition of some microbial groups, ultimately impairing ecological functioning and functional stability (direct effect) [84,85,86]. Likewise, contaminants may exert selective pressure on the genetic and phenotypic variability of microbial populations, potentially driving resistance development or altering physiological traits, such as contaminant degradation capacity (indirect effect). An example of the latter is the concept of Pollution-Induced Community Tolerance (PICT), which is based on the fact that contaminant exposure can shift communities towards more tolerant microbial species, thereby increasing overall tolerance [87,88].

A major challenge in AR research is linking bacterial hosts to identified ARGs in complex environments [18,26]. Establishing this connection is crucial for the evaluation of the ecological and clinical relevance of the detected ARGs and related risks. By identifying the dominant ARG-carrying bacteria, researchers can design targeted control interventions in order to limit the emergence and transmission of AR among environmental bacteria or, more alarmingly, to clinically important human pathogens. Moreover, the identification of host-range associations can enhance our understanding of the conditions driving the expansion or restriction of ARG host specificity across different environments. Linking ARGs and MGE-linked genes to their bacterial hosts provides valuable insights into potential primary disseminators of ARGs within complex microbial communities.

This issue is commonly addressed by computational approaches: (i) statistical inference from non-assembled metagenomic data comparing ARG abundances across samples with varying community compositions [18]; and (ii) assembly-based methods where short reads are reconstructed into longer overlapping DNA segments (contigs), drafted into metagenome-assembled genomes (MAGs), and scanned for associations between ARGs and phylogenetic markers [15]. However, approaches relying solely on correlation analysis of gene abundances offer some insights but are generally inconclusive [26,71]. Similarly, the reliable assembly of contiguous sequences encompassing ARGs from metagenomic shotgun data still poses significant difficulties [50]. Some attempts have been made to obtain ARG-host information directly from metagenomic short reads by pre-screening ARG-like reads [89]. Advances in long-read sequencing technologies are helping to overcome the limitations inherent to short-read methods, offering new opportunities to accurately link ARGs with bacterial hosts [23,33].

The Emulsion, Paired Isolation and Concatenation Polymerase Chain Reaction (EpicPCR) enables the linkage of a specific gene of interest to its host bacterium in a culture-independent manner [90,91]. Efforts have been made in recent years to overcome some of the constraints inherent to this technique [92,93], with promising results [94]. Furthermore, Fluorescence-Activated Cell Sorting (FACS) combines flow cytometry with cell sorting based on fluorescence, allowing for the identification of ARG-carrying bacteria tagged with fluorescent bioreporters or probes, which are later characterised via metabarcoding or metagenomic sequencing. Finally, the use of Chromosome Conformation Capture (3C) techniques, particularly Hi-C sequencing methods, has been extended to identify ARG hosts by physically cross-linking DNA in individual cells prior to metagenomic sequencing [95]. As with all methodologies, each of these approaches presents strengths and limitations. Readers interested in a more comprehensive technical evaluation are referred to Rice et al. (2020) [26].

### 3.5. Source-Tracking and Faecal Contamination Indicators

The identification of those sources potentially or actually affecting and regulating the composition of a given microbial community or resistome remains a major hurdle in environmental AR monitoring. Tracing the origin of ARGs is further complicated by the complex interplay of environmental drivers and the dynamic behaviour of AR genetic determinants. Much further research is needed to better understand the origin of both microbiomes and resistomes, since it is a crucial aspect for reducing ARG dissemination and developing effective AR mitigation strategies [96].

Recent progress in machine learning, combined with metagenomics, holds great promise for delivering more reliable solutions for microbial source tracking (MST). Notably, two novel tools have been developed for this purpose: (i) SourceTracker, which uses Bayesian theory and Gibbs sampling to infer the proportional contributions of different sources to sink samples based on community profiling [97]; and (ii) FEAST, that employs an expectation-maximisation algorithm to improve speed and accuracy [98]. Building on these foundations, modifications of these methods and alternative approaches have emerged, including Meta-SourceTracker [99], STENSL [100], EXPERT [101], and SNV-FEAST [102], though they remain less widely applied. While initially developed to unravel the origin of microbial communities, these tools have also proven effective in disentangling source-sink relationships of ARG profiles [96]. Multiple studies on freshwater ecosystems have applied them to investigate sources of both microbiomes [103,104,105,106,107] and resistomes [106,108,109,110,111,112,113]. However, caution is warranted when interpreting source proportions in ecologically dynamic environments, where microbial interactions influence community assembly [114].

In recent years, the connection between faecal contamination in aquatic environments and ARG abundances in human-impacted ecosystems has gained increasing attention [115,116]. Faecal contamination can arise from both point sources, such as wastewater treatment plants (WWTPs) and septic tank effluents, or diffuse sources, including agricultural runoff, due to faecal sludge being used as fertilizer, and livestock and wildlife defecation [116,117]. Both the risk associated with aging sewer infrastructures in some countries, which can lead to leaks and untreated discharges, and the increasing frequency of extreme weather events, including flooding, are forecasted to exacerbate the contamination of water resources [118].

The relationship with AR dynamics mainly stems from microbial loads and pathogens or ARB introduced via faecal inputs. These can alter bacterial community composition, diversity, and functional performance, promoting the selection and proliferation of resistant strains. In addition, faecal contamination directly contributes to ARGs and AR co-selective agents (e.g., metals, biocides, pharmaceuticals) that facilitate AR maintenance and HGT events [116,119]. Therefore, these inputs not only introduce readily detectable ARGs but may also affect the mobilization and persistence of ARGs already present in environmental microbiomes.

Traditionally, faecal contamination has been assessed by enumerating faecal indicator bacteria (FIB). However, despite its widespread application, this approach has inherent limitations, as it offers no information on source origin, pathogenicity, virulence, or resistance profiles [118,120,121,122]. To address these shortcomings, substantial efforts have been directed towards alternative markers for faecal source tracking (FST), with increasing emphasis on culture-independent techniques. Host-associated genetic markers for different animal sources have been developed and validated, enabling a more accurate identification of faecal inputs. Nonetheless, human-specific markers are particularly valuable, given that human faecal contamination generally poses a greater risk to public health than most non-human sources [120,123]. Particularly, bacteriophages, such as coliphages and *Bacteroides* phages, have been proposed as FST human-specific alternatives, though their normal low concentrations in diluted natural water systems can hinder detection. One promising candidate is *crAssphage* bacteriophage, identified some years ago [124] and reported as one of the most abundant viruses in human faeces and sewage wastewaters [125]. Due to its high prevalence and host specificity [126], *crAssphage* is now commonly used as a human faecal indicator.

Faecal contamination markers, long used to protect public health and regulate recreational and drinking water resources [117], have only recently been incorporated into AR research. Their inclusion now plays a growing role in identifying sources of AR determinants in freshwater ecosystems [107,109,110,119,127,128,129,130,131]. Expanding their application to environmental ARG monitoring programs could facilitate detection in systems impacted by faecal contamination and help distinguish hotspots of AR selection from receiving areas where the AR risk arises solely from the direct introduction of ARB or ARGs. However, in certain pristine environments with low ARG abundance, the correlation between ARG dynamics and faecal indicators may not always hold [10], highlighting the need to identify settings where this relationship is unclear or non-existent, in order to avoid inappropriate reliance on faecal markers. Furthermore, it remains uncertain to what extent faecal pollution correlates with latent ARGs [10]. Overall, a “toolbox approach” combining multiple markers, detection methods, and sampling strategies is widely advocated, recognising that no single indicator can conclusively characterise faecal contamination [118].

### 3.6. Contaminants as Drivers of Resistance

As important as assessing the prevalence and abundance of AR determinants and the structure and composition of microbial communities is the evaluation of selective pressures exerted by environmental contaminants in aquatic systems. While antibiotics are widely recognised as key selective agents for AR acquisition and spread, a growing body of evidence highlights the role of other emerging contaminants though co-selection mechanisms [77]. These include disinfectants and antiseptics [132], heavy metals [78], and pesticides (herbicides, fungicides, and insecticides) [133], among others. The presence of such contaminants, alone or in combination, may thus significantly influence the resistome in aquatic ecosystems, even in the absence of direct antibiotic exposure.

In this regard, several important considerations must be taken into account:

1. Most studies have focused on a limited number of selected contaminants present in aquatic environments. Targeted analytical methodologies provide reliable information about the presence and concentration of those compounds, even at trace levels. However, since analytes must be selected in advance, compounds not included in the contaminant target list remain undetected. In contrast, non-target and suspect screening methodologies allow for the simultaneous detection of a broad spectrum of compounds without requiring chemical standards until the confirmation stage [134,135]. Nevertheless, information on the structures and identities is only tentative and accurate quantification cannot be performed.
2. Transformation products and metabolites are frequently excluded from these analyses, despite their potential biological activity and contribution to AR selective pressures [136]. Transformation products arise from abiotic chemical or physical changes, while metabolites are generated through biological processes. Moreover, many compounds are excreted as conjugates, i.e., chemically bound to other molecules. Regardless of their origin, these novel chemical entities are of particular concern, as they may occur at higher concentrations than their parent compounds and may themselves exhibit pharmacological activity [136,137]. In some cases, they are also more persistent or toxic than the original substances. Interestingly, some studies have reported back-transformation processes that can convert such derivatives back to their parent chemicals under certain conditions [138]. In any case, their identification and quantification remain challenging due to the lack of analytical standards and incomplete reference databases [137]. Non-target analysis approaches hold promise for expanding the list of substances to be analysed.
  In the specific case of WWTPs, this consideration is especially relevant as some treatment processes also generate by-products [136,139]. Moreover, during biological treatment processes, certain conjugates may be metabolized and broken down, releasing the parent compound. This can result in higher concentrations in treated effluents, compared to influents, leading to apparent negative removal efficiencies [140].
3. The complex mixture of contaminants in aquatic environments often results in synergistic, additive, or antagonistic interactions that can modulate AR selective pressures in unpredictable ways. Therefore, evaluating contaminants individually may overlook combined effects that can be critical drivers of AR emergence and evolution. The wide variety of strategies available to study this “cocktail effect” complicates efforts to overcome this issue in standardised regulatory frameworks [141].

## 4. Where to Monitor?

### 4.1. Spatial Distribution of AR

Exploring the spatial distribution and variability of AR during its environmental monitoring is key for determining the origin and dispersal patterns of both chemical (antibiotic residues and their transformation products) and biological contaminants (ARB, ARGs, MGE-linked genes) associated to the AR problem. The identification of potential contamination sources and dispersion patterns enables a more informed selection of sampling locations and depths [142]. This consideration becomes particularly relevant in lotic systems, characterised by pronounced spatial complexities, with a longitudinal structure that extends from headwaters to the mouth and a lateral dimension encompassing the main channel to adjacent floodplains.

Spatial variability is largely governed by the type and location of contamination sources, which can generally be categorised as point or diffuse sources. Among diffuse sources, tributary inputs, urban runoff, and agricultural drainage are well-known for introducing contaminants in a variable and dispersed manner. Notably, runoff is considered one of the primary causes of surface water impairment, potentially carrying a wide range of contaminants depending on land cover (e.g., urban, cropland, forested areas) and land-use practices (e.g., agriculture, mining, recreational activities) [143]. In contrast, point sources, such as hospital and industrial discharges or WWTP effluents, typically lead to localised and elevated contaminant concentrations. Point sources are often easier to identify and regulate, yet their downstream impacts can be substantial, especially when compounded by diffuse contributions.

The hydrodynamic conditions of surface water systems strongly influence the spatial distribution of contaminants [142]. Flow dynamics shape the dilution, transport, and deposition of both antibiotics and ARGs, which in turn affect their environmental impact and dissemination patterns across aquatic landscapes [144,145]. Dissolved and particulate-bound contaminants may either be carried over long distances during high-flow events or accumulate in depositional areas during low-flow periods. Moreover, flow conditions control surface runoff and determine how contaminants are mobilised from surrounding urban, industrial, and agricultural areas into the receiving waters. Finally, flow regime fluctuations influence the lateral connectivity between the main waterbody and adjacent habitats (riparian zones, wetlands, etc.).

### 4.2. Sampling Design

The sampling design is inherently dependent on the objectives of the study and, specifically, on the questions it aims to answer. A well-structured sampling design is fundamental to the success of any environmental AR monitoring program. Once the objectives of the monitoring program, as well as the types of samples to be collected, have been defined, the sampling strategy can be developed [67]. A probabilistic or random sampling design, in which sites are selected at random, is more appropriate when the ultimate goal is to provide an unbiased assessment of the status of a given water body or to characterise AR baseline levels at a large scale. In contrast, a targeted or purposive sampling strategy, where locations are selected based on known or suspected issues, is more suitable for identifying potential drivers in high-risk areas. Likewise, in cases involving only a single localised source of contamination, a limited number of sampling points may be sufficient to obtain a general overview of the site-specific contamination. Instead, those sites characterised by complex contaminant distributions and dynamic hydrological conditions will require a higher number of monitoring locations, as well as multiple sampling points per location. The distribution of monitoring points should be sufficient to cover the study area and ensure an acceptable level of representativeness in the measurements. However, as abovementioned for method selection, the sampling strategy is often strongly constrained by financial limitations.

Decisions regarding sampling locations should be based, when available, on historical data and prior information. Accessibility and safety are also primary considerations when designing the sampling scheme, as certain site-specific conditions can influence sample collection and limit sampling feasibility. An interesting possibility is to include sampling locations already integrated into ongoing or planned surveillance programs. By incorporating environmental AR monitoring into already established monitoring infrastructures, coordination can be streamlined, while reducing costs and optimizing the use of available resources. Nonetheless, while such an approach is certainly worth of consideration, the adaptation of existing monitoring schemes to include specific markers of AR may prove complicated due to the scale at which it would need to be implemented [10].

Another approach is to focus on high-risk environments [5,10]. These settings are characterised as: (i) areas where humans are most exposed to ARB present at levels sufficient to colonise or infect humans; (ii) environments closely connected to both humans and animals; (iii) locations where selective pressure is exerted due to anthropogenic activities; and (iv) scenarios with the potential to act as vectors for the dissemination of ARGs. In this regard, WWTPs are key examples within freshwater ecosystems, as they harbour dense and complex bacterial communities, often including pathogens [5,146]. Moreover, since antibiotic concentrations in these environments are typically higher than in receiving waters, WWTPs may serve as significant hotspots for the evolution of AR [139,147,148]. Finally, water serves as a pathway for the dissemination of AR to both human and animal populations [27,149].

In studies on the impact of WWTPs on AR, it is crucial to include sampling points both upstream and downstream of the discharge site. Upstream sampling locations provide a reference for environmental conditions before the influence of the effluent outfall, thus allowing researchers to distinguish background levels from impacts associated with wastewater discharge. Reference sites should be located at a sufficient distance from the main contamination sources and, when human disturbances are widespread, the concept of least-disturbed conditions should be applied [150]. Downstream sampling points, on the other hand, capture the effects of effluent discharge on the receiving environment and, when selected farther from the discharge point, can also help assess the resilience of microbial communities to contamination-induced environmental impacts. It is important to analyse the specific context of the WWTPs under study, including particular circumstances and the nature of the water they receive [151]. Operational conditions, plant design, and the specific treatment processes and technologies applied in the WWTP can all significantly influence the presence, persistence, and spread of ARB and ARGs [152]. The processes taking place within the water treatment system itself can be evaluated by analysing influent and effluent samples or by collecting other samples at key stages of treatment.

### 4.3. Sample Matrix Selection and Relevance of Understudied Habitats

Various environmental compartments have been recognised as important reservoirs of ARB and ARGs in freshwater ecosystems. The selection of the sample matrix and habitat type is a key aspect to take into consideration in environmental AR monitoring programs, as it can influence ARG detection and data interpretation. Different environmental matrices vary in their capacity to accumulate, preserve, or dilute genetic material (e.g., ARGs), thereby reflecting different levels of exposure risk or ecological significance. Thus, water samples might represent more transient or diluted “signals”, whereas sediments or faecal materials can serve as reservoirs of persistent ARGs. To capture this variability, effective monitoring networks should encompass a range of environmental matrices, and provide a coherent and comprehensive overview of both their ecological and chemical status. This approach helps ensure that no critical reservoir is overlooked and supports a more accurate assessment of environmental risks associated with AR.

The water matrix has traditionally been the primary focus of freshwater environmental studies and surveillance efforts, due to its accessibility and central role in the hydrological cycle. Planktonic communities, in particular, are widely used as indicators of water quality because of their direct susceptibility to changes in water conditions. Moreover, water serves as a vector for the dissemination of AR between natural environments and human and animal populations [27,149], as well as between different water systems [153]. This emphasis is reflected in the existing regulations, with water being extensively monitored and regulated under various legal frameworks. However, in the context of environmental AR, while being critical for the transport of ARB and ARGs, the water matrix is probably a less significant reservoir of ARGs compared to more stationary environmental matrices [154]. Still, in certain settings, such as those impacted by wastewater effluents, water remains an important matrix to monitor for AR emergence and dissemination. Studies on river ecosystems have shown that the resistome of effluent-receiving waters often closely resembles that of the discharged wastewater and, although not typically classified as a sink for AR determinants, river water is frequently the matrix most directly affected by wastewater inputs [155].

Also, WWTPs themselves represent an ideal setting for the collection of material relevant to AR monitoring. Wastewater-based surveillance (WBS) has emerged as a valuable approach for monitoring a wide range of public health indicators, including AR determinants and human pathogen infections. This approach has emerged as an alternative method to conventional clinical surveillance of AR since wastewater samples can provide information from whole human populations served by the sewage network [156,157,158]. Moreover, WWTPs are typically equipped with sampling devices for the routine collection of environmental compliance and quality control samples. Furthermore, particularly since the COVID-19 pandemic, the potential of wastewater analysis for the early detection of health risks and community-level health surveillance has been highlighted [159,160]. Therefore, the integration of WBS into existing environmental AR monitoring frameworks can strengthen existing early warning systems and support more effective public health responses. By analysing wastewater and other sample types (e.g., influent, effluent, sludge), WBS would enable the detection and quantification of ARB and ARGs circulating within human populations.

The significance of aquatic sediments has also been widely recognised over the past decades, prompting improvements in analytical methods and protocols [161]. The sediment matrix not only acts as a vector for contaminants but also serves as an important sink compartment for them [162]. Aquatic sediments contain a great variety of organic matter sources, making them a suitable habitat for a wide range of (micro)organisms with varying nutrient requirements [84,154,163]. Microbial communities inhabiting aquatic sediments often form a very complex and highly diverse assemblage of prokaryotic and eukaryotic organisms. Similarly, sediments commonly exhibit a high pore-scale heterogeneity and the presence of anaerobic microsites [164], which foster a greater abundance of narrower phyla and/or unknown taxa. Relevantly, different studies have highlighted the direct role of aquatic sediments as reservoirs of ARGs [162,165,166]. Indeed, a growing body of literature reports the abundance and diversity of ARGs in riverbed sediments, as well as in the sediments of other freshwater systems. Free DNA, including ARGs, generally persists longer in the sediment matrix than in the water matrix, as sediment and clay particles adsorb DNases that would otherwise hydrolyse the extracellular DNA [163,167,168,169,170]. As a result, DNA can persist in aquatic sediments for months to millennia [171], resulting in the preservation of ARGs in the environment long after the corresponding AR selective pressure has been removed.

Additionally, the significance of environmental biofilms and periphytons is frequently highlighted, as they represent the preferred lifestyle of bacteria in natural environments [172]. These structured multicellular communities are embedded in a self-produced matrix of extracellular polymeric substances. In aquatic habitats, biofilms develop not only on solid benthic substrates, such as riverbed pebbles and sand (epilithic and epipsammic biofilms, respectively) but also at air-liquid interfaces as floating macro and microaggregates (floating and pellicular biofilms) [173,174]. From an ecological perspective, microorganisms in environmental biofilms actively participate in the decomposition of organic matter, nutrient dynamics, and biogeochemical cycles, acting as key components of ecosystem functioning. River biofilms can account for up to 90% of the total microbiota (prokaryotes, algae, fungi, protists) and are important sources of primary production. Their exposure to toxic contaminants can lead to bioaccumulation and/or biomagnification problems [175]. This fact, along with their relatively rapid growth, the temporarily sessile nature of their microbial communities, and their contextual dependence on the physical and chemical conditions of the surrounding environment, makes biofilms trustworthy biosensors of overall water quality and the ecological status of freshwater ecosystems [176,177].

Environmental biofilms, often found to harbour higher abundances of ARGs compared to other aquatic matrices [154,178,179,180], have been recognised as peripheral compartments with key importance as long-term AR reservoirs [162,181,182], most likely due, at least in part, to their greater survival and resistance to environmental and chemical stressors. The bacterial cells that make up biofilms can display 10 to 1000 times lower susceptibility to specific antimicrobial agents, compared to their planktonic counterparts [183,184]. This reduced susceptibility results from a combination of factors, namely: (i) the protection provided by the extracellular polysaccharide matrix, which limits the penetration of antimicrobial agents; (ii) the selection of resistant bacteria promoted by sublethal concentrations of antimicrobials that manage to penetrate the biofilm; and (iii) the presence of persister cells, dormant, or slow-growing cells that display temporary resistance phenotypes and can trigger stress responses under unfavourable chemical conditions [174,176,185,186]. Biofilm formation can indeed be a defensive reaction to the presence of antimicrobials [176].

The relevance of biofilms in AR can also be attributed to a higher genetic transfer efficiency [173,187]. Several factors increase the likelihood of genetic exchange within these structures: (i) high cell density, close contact, and restricted bacterial motility within the biofilm matrix, promoting bacterial interactions; (ii) increased genetic competence in a polymicrobial environment, acting as reservoirs of genetic diversity; and (iii) accumulation of MGEs or free extracellular DNA released through cell lysis or active secretion systems. Moreover, ARB that detach from biofilms can disperse into the environment potentially posing a threat to AR spread [173]. Finally, several studies have reported the potential accumulation of pharmaceuticals in biofilm matrices [178,188,189], which may then act as AR selective pressure agents.

In consequence, biofilms are often considered hotspots for the acquisition and spread of AR. Their impact on public health is particularly significant when we take into consideration that biofilm formation is a common feature of many bacterial pathogens. Actually, many chronic infections are linked to biofilm growth on natural surfaces (e.g., teeth, lungs) and medical devices (e.g., pacemakers, catheters, prosthetic heart valves) [186,190]. Several authors have thoroughly reviewed clinically relevant biofilms, but much less is known about the role of environmental biofilms as natural reservoirs of AR. It is important to emphasise that the effect of antibiotic exposure on multispecies environmental biofilms can differ significantly from that observed in single-species clinical biofilms [174,176].

Despite the considerations outlined above, most studies still focus on a single matrix, and only a few have incorporated what are considered understudied habitats. Matrices such as aquatic fauna guts (amphipods [155], fishes [154,191,192,193]), and detritus [154], among others, can be important reservoirs of AR but remain largely unexplored. Abramova et al. (2023) [11] scrutinised the PubMed database for publications containing relevant qPCR data on ARGs in environmental samples and concluded that water, faeces, and sediments were the most commonly studied sample types.

In the context of AR environmental monitoring, highly relevant matrices, such as stream biofilms, have only recently begun to receive attention. Table 4 presents a variety of field studies evaluating AR dynamics in stream biofilms under natural conditions (excluding microcosm and mesocosm systems). Only biofilms collected from aquatic environments are included, excluding those from drinking water or water treatment systems (such as pipes and similar structures), as well as those associated with the so-called “plastisphere”. As shown in Table 4 (see also Table S2 in Supplementary Material for further methodological details), there is a notable research gap regarding the antibiotic resistome of natural river biofilms. Most biofilm studies focus on changes in the structure and composition of their microbial communities, with few investigating the antibiotic resistome within them and even fewer comparing sessile communities to their planktonic counterparts. Additionally, existing research on antibiotic resistomes typically targets a limited set of genes using qPCR or, at best, high-throughput qPCR approaches. It is only in recent years that some studies based on metagenomic techniques have started to appear.

**Table 4:** Field studies evaluating antibiotic resistance in stream biofilms under natural conditions.

Location	Freshwater System	Sampling Campaigns	Sample Collection *	Methodological Approaches *	References
Austria	Three tributaries (Traisen- Gölsen, Ybbs, Kamp) and short stretch of Danube River, upstream and downstream municipal WWTPs	Five occasions in October 2020, January 2021, April 2021, July 2021, October 2021	Rock or wood branches scrubs	qPCR analysis; 9 genes: *sul1*, *tetM*, *qnrS*, *bla_TEM_*, *bla_KPC_*, *bla_CTX-M-1_*, *bla_CTX-M-9_*, *bla_OXA-48_*, *intI1*	[194]
Spain	Onyar River affected by a secondary treated wastewater effluent	Year-long period during autumn, spring, and summer	Scrubs from randomly selected streambed cobbles	qPCR analysis; 7 genes: *sul1*, *tetM*, *qnrS*, *bla_TEM_*, *bla_OXA-58-58_*, *bla_CTX-M-32_*, *intI1*	[195]
China	Heihui River, encompassing densely populated urban areas, farmland, industrial and mining zones, forests	One sampling campaign (May 2022)	Scrubs from rocks at a depth of 15–30 cm along the riverbank	Shotgun metagenomic sequencing; Novaseq 6000 platform, paired-end (2 × 250 bp) strategy	[196]
United States	Scioto River watershed including Scioto River, Olentangy River, and Big Darby Creek	Between October 2017 and August 2018 in 4 visits (autumn, winter, spring, summer)	Rock scrubs	Oxford Nanopore Technology’s, long-read MinION	[197]
United States	Raritan River, sites with varying influences by wastewater effluent, urban activities, agricultural activities, and tides	Not reported	Rock and leaves scrubs (8 × 16.5 cm^2^)	qPCR analysis; 2 genes: *sul1*, *vanZ*, and 16S rRNA gene	[198]
Brazil	Guaporé River watershed, including Capingui River, Marau River, Lajeado-Carazinho River, and Lajeado River	Beginning of summer (December 2014) and winter (June 2015)	Scrubs from rocks that remained submerged in all seasons	qPCR analysis; 3 genes: *sul1*, *qnrA*, *erm* and 16S rRNA gene	[199]
Germany	Holtemme river; upstream and downstream a WWTP	Dry period in summer 2022, five sampling days	Scrubs from water-facing side of riverbed stones	qPCR analysis; 3 genes: *sul1*, *sul2*, *intI1* and 16S rRNA gene	[200]
Switzerland	Sampling sites encompassing WWTPs and upstream and downstream sampling sites in receiving rivers	Between July and October 2017	Rock scrubs	Shotgun metagenomic sequencing; HiSeq 4000 System (Illumina), paired-end (2 × 150 bp) strategy	[155]
Various regions globally (4 countries)	Rivers, lakes, streams, caves, and other environments (see Supplementary Information in publication)	See Supplementary Information in publication	See Supplementary Information in publication	See Supplementary Information in publication	[201]
China	Lung Fu Mountain stream and Sam Dip Tam	One sampling campaign (April 2018)	Scrubs from benthic rocks of identical size in similar flow conditions	Metagenomic approaches: BGISEQ-500 platform	
France	Poitiers WWTP and upstream, and downstream sampling sites in Clain river	One sampling campaign from January to December 2018 (each month)	5-month river-incubated sterile rocks, pooled as a single bulk sample	qPCR analysis; class 1, 2 and 3 integrons and 66 ARGs, 5 multidrug efflux pumps, 6 MRGs, 3 disinfectant resistance genes, 11 MGEs	[202]
United States	Scioto River, Olentangy River, and Big Darby Creek; sampling sites: outflow upstream, outflow, outflow downstream, left and right bank	3–4 times from October 2017 to August 2018	Rock scrubs, six samples collected at each sample site	ddPCR analysis; 3 genes: *bla_KPC_*, *bla_NDM_*, *bla_OXA-48_*	[154]
Germany	Kraichbach River; WWTP upstream and downstream sampling sites	Five sampling campaigns from February to June 2019, once a month	Biofilm samplers: PVC box with four 70 cm × 30 cm glass sheets; submerged for ~1 month	qPCR analysis; 12 genes: *bla_TEM_*, *ermB*, *tetM*, *sul1*, *bla_CMY-2_*, *bla_CTX-M_*, *bla_CTX-M-32_*, *bla_OXA-48_*, *mecA*, *bla_NDM-1_*, *bla_KPC-3_*, *mcr-1*	[180]
United States	Three streams in Cuyahoga River watershed including Tinkers Creek, Yellow Creek and Furnace Run	4 campaigns: November 2012, April 2013, June 2013, August 2013	Scrubs from cobble-sized stones	qPCR analysis; 7 genes: *tetW*, *sulI*, *sulII*, *pbrT*, *copA*, *czcA*, *czcC*	[203]
China	Bosten Lake and Ebi Lake	Not reported	Algae and wood biofilms	qPCR analysis; 2 MGEs (*intI1*, *ISCR1*) and 20 MRGs	[204]
China	Yangtze Estuary	Sampling campaign in October 2016	Scrubs from the surface of submerged civil engineered cement structures and rocks	PCR and qPCR analysis; 22 genes: *sul1*, *sul2*, *sul3*, *sulA*, *qnrS*, *qnrB*, *aac(6′)-Ib*, *tetA*, *tetB*, *tetC*, *tetE*, *tetG*, *tetL*, *tetM*, *tetO*, *tetQ*, *tetS*, *tetT*, *tetW*, *tetX*, *ermB*, *Chl*	[178]
Spain	Two tributary streams to the Ebro River, including Montsant stream and Matarranya stream; sampling sites upstream, discharge point, and downstream UWWTP	Not reported	Epilithic biofilms: rock scrubs, and epipsammic biofilm: streambed top layer fraction (0–5 cm)	qPCR analysis; 13 genes: *bla_TEM_*, *bla_CTX-M_*, *bla_KPC_*, *bla_NDM_*, *bla_OXA-48_*, *qnrS*, *sul1*, *sul2*, *tetM*, *tetW*, *ermB*, *vanA*, *intI1*	[205]
Spain	Two tributary streams to the Ebro River, including Montsant stream and Matarranya stream; sampling site downstream UWWTP	Not reported	Samples collected in triplicate in Eppendorf tubes	qPCR analysis. All known alleles of *bla_KPC_*, *bla_NDM_*, and *bla_OXA-48_*-like genes	[206]
France	Vienne River watershed and their WWTPs; sampling sites: upstream, downstream (‘0 m’, ‘10 m’, ‘100 m’)	Three consecutive days in July 2011	Scrubs from 5–10 rocks collected randomly and submerged 50–100 cm all over the year	qPCR analysis; Class 1, 2, and 3 integrons (*intI1*, *intI2*, *intI3*)	[175]
New Zealand	Four watersheds: Waiau, Aparima, Oreti, Makarewa, land uses including pasture farming, cropping, forestry, native grasslands, indigenous forest, government-managed conservation land, sparsely populated townships	4-day samplings: July 2010, August–September 2010, October 2010, December 2010, January 2011, March 2011, April 2011, May 2011	Scrubs from three randomly collected rocks of roughly 10 cm diameter and likely continuously submerged	PCR analysis; 10 genes: *aacA*-*aphD*, *mecA*, ermA, *ermB*, *tetA*, *tetB*, *tetK*, *tetM*, *vanA*, *vanB*	[207]
Spain	Tordera River Basin, including Gualba stream, Repiaix stream, Xica stream, Tordera stream and 3 WWTPs, including Gualba, Breda and Arbúcies plants	Not reported	Scrubs form a 50–100 m stream section; cobble surface area estimated via weight/area regression	qPCR analysis; 4 genes: *bla_CTX-M_*, *qnrS*, *sulI*, *ermB*	[208]
Spain	Ter River; sampling sites upstream and downstream Ripoll WWTP	Two sampling events: June and September 2010	Rock scrubs	PCR analysis; 4 genes: *qnrA*, *qnrB*, *qnrS* and *aac(60)-Ib-cr* for ciprofloxacin-resistant isolates. qnr-positive isolates investigated for *bla_CTX-M_*, *bla_SHV_*, and *bla_TEM_*	[209]
New Zealand	Taieri River, land uses including livestock farming, cropping, market gardening, forestry, native grasslands, and sparsely populated townships	Year-long duration	Scrubs from three randomly collected rocks of roughly 10 cm diameter and likely continuously submerged	PCR analysis; 10 genes: *vanA*, *vanB*, *mecA*, *ermA*, *ermB*, *tetA*, *tetB*, *tetK*, *tetM*, *aacA-aphD*	[210]
Spain	Ter River; sampling sites upstream, discharge point and downstream Ripoll WWTP	June 2010, end of spring	Rock scrubs, samples collected in duplicate	qPCR analysis; 11 genes: *bla_TEM_*, *bla_CTX-M_*, *bla_SHV_*, *qnrA*, *qnrB*, *qnrS*, *tet(O)*, *tet(W) sul(I)*, *sul(II)*, *erm(B)*	[165]
Australia	Mars Creek, small urban watercourse with no hospitals, sewage treatment works, or animal production facilities	Not reported	6 biofilm samples	PCR analysis; integrons carrying *qac* gene cassettes	[211]

* In “Sample Collection” and “Methodological Approaches” columns, only information specifically related to biofilm and quantification of AR dynamics is included.

## 5. When to Monitor?

### 5.1. Chemical Contamination Temporal Trends

Temporal patterns in contamination levels play a crucial role in understanding AR dynamics. Monitoring the presence of antibiotics and other emerging contaminants provides valuable context for interpreting AR data and allows the identification of correlations between variations in contaminant loads and AR patterns over time. The identification of temporal trends has become more crucial than ever in the current scenario of climate change, where pronounced shifts in precipitation patterns and extreme temperatures are increasingly common [145,212]. In the specific case of the impact of WWTP effluents, the identification of temporal trends is essential when assessing their contribution to the load of ARGs in receiving waters [213]. The same applies to other event-based contaminations or resistance inputs [214], as well as systems with flow regulation mechanisms (e.g., dams or sluice gates) [168].

Temporal trends in freshwater ecosystems are often associated with the hydrological characteristics of these water systems. Fluctuations in flow rates directly affect contaminant solubility, as well as partitioning and adsorption rates between the water phase and sediments [215]. Water scarcity during drought periods make freshwater ecosystems particularly vulnerable to the impact of contaminants due to decreased solubility rates [216,217]. In contrast, during high flow conditions, it is crucial to consider the increased surface runoff and transport of contaminants, which may lead to contamination from nearby sources or upstream sites, respectively [144,145,218].

In river systems, it is necessary to take abiotic and biotic contaminant degradation processes into consideration. Temperature is a key factor influencing contaminant degradation rates and microbial activity in surface waters [219]. During the summer months, higher temperatures often enhance the biodegradation capacity of bacterial communities [220,221], while increased sunlight exposure may trigger photochemical degradation processes [222,223]. At the same time, low-flow conditions during summer periods result in faster photochemical reactions due to efficiently illuminated shallower waters and decreased flow velocities [224]. By contrast, during high-flow circumstances, water turbidity may result in increased stability of some compounds [144].

**Figure 3:**
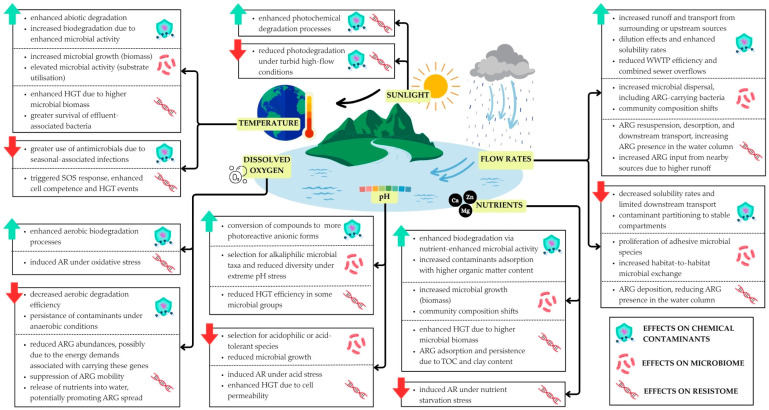
Influence of environmental parameters on chemical contamination, resistome, and microbiome profiles, highlighting their role in shaping temporal trends and informing strategic decisions on when to monitor in order to achieve effective AR surveillance. Green upward and red downward arrows indicate an increase and a decrease in environmental parameters, respectively. AR: antibiotic resistance; ARG: antibiotic resistance gene; HGT: horizontal gene transfer (created with https://www.canva.com (accessed on 23 July 2025)).

Contaminant biodegradation rates result from a complex interplay of various environmental factors (Figure 3) which can directly and indirectly influence the capacity of microbial communities to degrade contaminants. Biodegradation rates have been linked to different water parameters, such as pH, dissolved oxygen (DO), and dissolved and particulate organic matter [219,225]. In particular, pH can alter the speciation of ionisable chemicals and, consequently, modify their bioavailability and, hence, biodegradability [219]. Moreover, pH can affect photodegradation rates. When pH is higher than the dissociation constant (pKa) of a given contaminant, the anionic form predominates, and these forms tend to be more reactive under sunlight conditions [226]. The degradability of many substances also depends on their solubility and adsorption capacity to organic matter, both of which are ultimately influenced by other environmental factors (e.g., flow conditions, temperature, pH, sediment particle size, organic matter content, textural characteristics) [227,228]. It is important to note that many contaminants can disrupt the metabolic activity of microorganisms, further complicating the situation by impairing their degradation capabilities [219,229]. In any case, natural attenuation processes in river waters are largely determined by the specific physicochemical properties of each contaminant [215] and, then, their dynamics should always be assessed on a case-by-case basis.

Environmental factors also affect contaminant removal efficiencies in conventional wastewater treatment processes, given that biodegradation and sorption are the predominant removal mechanisms in wastewater treatment systems and are both temperature-dependent [230]. Moreover, the negative effect that rainwater can have on the removal efficiency of wastewater treatment systems is not negligible, leading to a reduction in hydraulic retention time, as the system has to process more water [139]. Also, increased precipitation can intensify combined sewer overflows (they collect both wastewater and rainwater in the same system) and wastewater bypass [139,214,231].

Finally, the occurrence of contaminants can also correlate with product consumption patterns in local human populations. In the specific case of pharmaceutical products, this is closely linked to prescription preferences among countries [144]. Climate change also plays a fundamental role, as emerging infectious diseases can alter the demand for certain medications and other products within the healthcare system and pharmaceutical industry [212,232]. Data on prescription habits or sales figures offer valuable insights into the substances likely to be detected in the studied area, facilitating more efficient design and planning of monitoring campaigns [142]. Unfortunately, this practice is uncommon, either due to the absence of such data being collected by public authorities or to technical limitations in the number and type of compounds that can be analysed by the corresponding research group.

### 5.2. Resistome and Microbiome Temporal Trends

When evaluating the temporal dynamics of ARG abundances, it is essential to consider not only contaminant (e.g., antibiotics) temporal trends. In the specific case of ARGs, temporal variations are not solely dependent on the selective pressure exerted by antibiotics and other emerging contaminants. The persistence of ARGs, even in the absence of such selective pressures, may be influenced by other potential driving mechanisms: (i) sub-inhibitory concentrations can still induce/promote the enrichment of ARB; (ii) ARGs may continue to disseminate through HGT events or co-selection mechanisms; and (iii) since ARGs confer adaptive advantages, bacteria carrying them may perform certain intrinsic functions more efficiently [83,233]. Furthermore, bacterial community dynamics can significantly influence ARG profiles, with both the composition and diversity of such communities being shaped by seasonal variations.

Flow rates inherent to each hydrological period not only affect contamination levels, but also directly impact microbial communities and their genes. During low-water conditions, there may be a deposition of ARGs into more stable/static environmental compartments (e.g., sediments, biofilms), reducing ARG abundance in the water column [234]. Likewise, a decrease in flow rate can enable the proliferation of adhesive species in these matrices and may favour microbial exchanges among different habitats [235]. In contrast, during high-water periods, desorption, resuspension, and downstream transport of ARGs may occur, along with the direct input of AR genetic determinants from nearby sources [108,169,213,234,236]. Notably, within the broader context of climate change, prolonged dry periods preceding rainfall events can lead to increased accumulation and subsequent release of ARGs into river water [237], and may differentially affect intracellular and extracellular ARGs [238]. Furthermore, during high-flow water periods, microbial aggregates including ARG-carrying bacteria may enter water bodies via runoff or be transported attached to suspended solids [239], often explaining shifts in microbial diversity and composition [108,235,237,240,241]. Similarly to chemical contaminants, the increased water turbidity and concentration of suspended solids found during high-water periods might favour the presence and spread of ARGs by reducing photodegradation under conditions of lower sunlight and UVA radiation [180]. This underscores the necessity of integrating both spatial and temporal perspectives when evaluating ARG dynamics, and highlights differences in water column-to-sediment/biofilm ARG ratios in the dry versus wet season.

Water temperature is also an important factor driving bacterial community dynamics [241,242,243]. Several authors have shown that higher temperatures can lead to increased microbial growth rates and biomass [218,244], which are reflected in 16S rRNA gene abundances [168,245]. Notably, a higher bacterial biomass may favour HGT transfer events due to increased presence of potential donor strains interacting with suitable recipients, thereby increasing the risk of AR dissemination [81,246]. Moreover, warmer water temperatures have been suggested to enhance bacterial persistence in the presence of antibiotics [247] and may also promote the survival of effluent-associated bacteria [246]. Temperature has been linked to enhanced microbial activity and variations in carbon substrate utilisation patterns, associated with increased metabolic rates [242,244,248]. Nevertheless, some authors have reported opposing trends with respect to temperature [245,249], possibly due to the fact that cold temperatures can act as environmental stressors, potentially triggering the SOS response and fostering AR by increasing cell competence, genetic recombination rates, and HGT events [245]. Moreover, this could be related to the higher persistence of ARGs observed under lower temperature and irradiance conditions [250,251].

In aquatic systems, seasonal variations in microbial community composition and biomass have been linked to changes in nutrients loads [240,242,243,252]. This is particularly relevant in situations involving anthropogenic allochthonous inputs [244], such as WWTP outfall, or conditions that accelerate the delivery of terrestrial dissolved organic matter which are themselves influenced by hydrological dynamics [253,254]. In a study by Di Cesare et al. (2017) [237] on the effects of rainfall events, a statistically significant relationship was found between precipitation intensity and the absolute and relative abundance of ARGs, total phosphorus, and ammonium nitrogen. This further highlights the interplay of environmental factors [84], such as, for instance, flow rates and nutrient loads. In turn, nutrient limitation and starvation can also induce AR, due to the stress they generate [251,255], in a manner similar to temperature effects. This fact may not be solely related to bacterial community dynamics, but may instead result from a direct effect on ARGs themselves. As above-mentioned, some authors have suggested that total organic carbon, in combination with clay, may exhibit strong adsorption capacity for ARGs in sediments, thereby protecting them from nuclease degradation [163,167,168,169,170] and contributing to downstream transport of ARGs during high-water periods.

The isolated effect of pH in freshwater ecosystems has been studied less frequently, showing varied effects on microbial diversity and ARG abundances [84]. Water pH has been found to shape bacterial community structure, affecting not only microbial diversity by imposing physiological stress but also influencing bacterial community assembly processes through environmental filtering [240]. Some studies have reported that a pH closer to neutral may represent more favourable conditions for microbial growth [245]. Regarding its effects on ARG profiles, pH may also influence the presence and mobility of heavy metals and other contaminants, thereby indirectly affecting ARG abundances [256]. However, some studies have also reported a direct acid stress-induced AR [251,257,258], attributed to pH homeostasis mechanisms through which bacteria regulate intracellular and extracellular pH under acidic conditions [259]. Acid stress responses include (i) modification of membrane channel size; (ii) proton efflux by H^+^-ATPase mechanism; and (iii) activation of proton pumps. These mechanisms play a critical role in AR development by contributing to antibiotic efflux and/or the alteration of antibiotic targets [251]. At low pH, the increased availability of protons can enhance the proton motive force, essential for driving antibiotic efflux mechanisms, but the effectiveness of the efflux pump inhibitor can also be affected [260,261]. Additionally, acid stress leads to alterations in membrane fluidity by modifying the bilayer structure, which can ultimately compromise cell viability. Nonetheless, these changes may also protect bacteria by reducing the permeability to acids and antibiotics [251]. Stress responses can lead to other physiological adaptations, such as stress-induced slow growth, dormancy, or entry into a persister state [257].

Dissolved oxygen has also been associated with AR, primarily due to its connection with oxidative stress responses. Previous studies have shown that DO is involved in the generation of reactive oxygen species (ROS), which can ultimately affect the activity and lethality of bactericidal antibiotics within the cell [262]. This phenomenon is sometimes linked to the activation of efflux pumps and alterations in membrane components (lipopolysaccharides) [251]. Likewise, when hydroxyl radicals come into close contact with DNA, they can cause strand breaks and subsequent cellular DNA damage. Depending on the source and extent of this damage, specific oxygen-responsive genetic and cellular machinery is activated to facilitate repair. In particular, induction of the SOS response can lead to the activation of error-prone polymerases, which allow replication to continue despite damage, but also increase the mutation rate promoting genetic modifications and rearrangements that contribute to the emergence of AR [262,263]. Also, sub-inhibitory concentrations of antibiotics have been reported to generate ROS, further exacerbating this process [263,264,265,266]. Several studies have investigated the influence of DO on bacterial community structure, both in laboratory bioreactors [262,267,268] and in the natural environment [269]. Laboratory-scale experiments have shown distinct ARG profiles under varying DO conditions (hyperoxic, normoxic, hypoxic), often reporting lower ARG abundances and potentially reduced ARG mobility in oxygen-limited environments [262]. Similarly, field studies have found a negative correlation between DO levels and ARG abundances, possibly due to energy demands placed on bacteria harbouring the ARGs under low-oxygen conditions [269]. Conversely, low DO levels may trigger nutrient release into the water column, which could in turn facilitate the spread of ARGs [269].

It is important to also consider that many of these environmental factors can similarly affect the removal rates of abiotic and biotic contaminants within WWTP facilities. Some studies have examined the potential effects of WWTP changing operational parameters and other factors related to climate change or seasonal variations [270,271,272,273,274,275,276], as well as extreme weather conditions [277], on contaminant removal rates. Challenges related to WWTP performance are often associated with changes in microbial density in the influent, largely driven by seasonally dependent parameters such as temperature. However, variations in contaminant dilution between wet and dry seasons can lead to operational difficulties, particularly during periods of low precipitation, when higher concentrations of contaminants may enter the treatment system. Nonetheless, as previously mentioned, these issues could also be linked to a reduction in hydraulic retention time [139], as well as to the occurrence of combined sewer overflows and wastewater bypass [139,214,231].

### 5.3. Fragmented Evidence and Incomplete Insights

Capturing temporal and seasonal variations remains challenging in environmental AR monitoring programs, since most of them rely on grab (active) sampling methodologies, where discrete samples are collected at a specific time and location. This sampling strategy is straightforward and cost-effective, but it only provides a snapshot of the conditions at that particular moment. In contrast, spatially, flow-, and time-weighted strategies, including both active composite and passive sampling methods, offer an averaged measure of contamination over larger spatial scales and longer periods, accounting for fluctuations in flow [28,278]. Nonetheless, the relatively high cost and logistical challenges associated with these strategies have led to their limited application. Moreover, some barriers, including sampler calibration requirements and hydrodynamic control issues, still prevent regulatory acceptance and the widespread implementation of these strategies for routine monitoring of contaminants in aquatic systems [137,142].

The grab sampling strategy frequently does not capture the full range of temporal changes and fails to represent those analytes and concentrations that fluctuate significantly over time [137,278,279]. This is particularly relevant in the context of wastewater, where intra-day (diurnal) variation in the concentration of specific contaminants is well documented, and hourly composite samples are recommended [137]. Similarly, some studies have reported taxonomic and resistome differences among methodologies used for collecting influent wastewater samples, with single-timepoint grab samples potentially overlooking clinically relevant ARGs that are better represented in composite sampling strategies [280]. Sample collection strategies can also influenced ARG concentrations and calculated removal rates in WWTPs [281]. Finally, composite samples have been associated with increased diversity of *Escherichia coli* isolates, compared to grab samples in hospital effluents [282].

Despite the inherent limitations of spot-sampling strategies, increasing sampling frequency and incorporating a high degree of replication can help mitigate some of these drawbacks and provide valuable insights into spatial and temporal variations of abiotic and biotic contaminants [279]. In any case, a grab sample is certainly better than having no sample at all. On the other hand, a major limitation of 24 h-composite sampling is the potential chemical instability of certain chemicals [137]. Moreover, while certain sample types may exhibit pronounced temporal variations, that is not always the case. Previous studies has reported relatively stable levels of AR over time, with no marked periodic fluctuations, further supporting the utility of single-timepoint sampling strategies in certain contexts [283]. Cornman et al. (2018) [284] reported that bacterial community composition remained consistent across sampling methodologies, and taxon-specific detection rates exhibited minimal variation.

Beyond these constraints posed by specific sampling approaches, our understanding of the temporal dynamics is typically restricted to a single season, or to comparisons between broad seasonal contrasts such as spring vs. autumn, summer vs. winter, or any of these combinations [168,235,244,246,252,285,286,287] and dry vs. wet (sometimes, vs. normal) conditions [234,236,240,254,288]. In a changing climate scenario, however, seasons commonly become irregular, meaning that such studies may not fully capture seasonal dynamics, particularly variability throughout the year, which would require a systematic, year-round, and high-frequency sampling [289,290]. Increasing recognition of the complex interplay between climate change and AR underscores the need for coordinated efforts to tackle these interconnected global challenges [291,292].

As demonstrated in previous studies [166,235,242,287,290,293], many changes can also be matrix-dependent. Some factors may exert differential effects depending on the specific environmental compartments, which may, in turn, exhibit varying levels of temporal responsiveness [84,234,287,290]. As a result, greater differences between matrices may arise depending on the time of year [287]. These patterns have been linked, for example, to a lower exchange rate between habitats during high-flow conditions or the concentration effect caused by reduced river water volumes during low-water periods [287]. The effect of flow rates, velocities, and heterogeneities is particularly relevant for biofilms, affecting both the diversity and relative proportions of biofilm bacteria [294,295,296]. Similarly, river flow conditions strongly influence sediment transport mode and rate [297]. In sediment and soil matrices, moisture content has been identified as an important environmental constraint on microbial biomass [244] and connectivity among microorganisms [298]. The effect of sunlight, influenced by the riparian canopy, can affect microbial functioning in a manner comparable to temperature [242,244], being particularly relevant for shaping benthic microbial diversity [299].

The inconsistent findings described above often reflect the complexity and variability of natural ecosystems. Predicting microbial community composition, and hence AR, based on environmental factors may be particularly challenging in stream and river ecosystems due to a highly variable hydrology [84] and greater temporal variability in community dynamics, compared to other habitats [300].

## 6. Conclusions

The emergence, spread, and evolution of AR in environmental matrices is currently a matter of much concern and interest, owing in great part to the existence of potential links between the environmental resistome and the human resistome. In this respect, freshwater ecosystems are nowadays considered critical sites for environmental AR, pointing to the imperative need to comprehensively monitor and study their potential role in AR development and dissemination. This review aimed to present a roadmap for designing and establishing environmental AR monitoring programs in freshwater ecosystems, framed around four essential questions (how? what? where? when?). By synthesizing current knowledge and methodologies, this review provides a practical framework that consolidates existing approaches, highlights critical considerations for implementation, and serves as a guide for researchers, regulatory authorities, and decision-makers in the planning and management of effective environmental antibiotic resistance monitoring programs. It was concluded that, due to the complexity, spatial heterogeneity, and temporal dynamic nature of freshwater systems, a lot of research is still needed to properly monitor and rightly evaluate, in both qualitative and quantitative terms, the potential risks derived from the occurrence and spread of AR determinants in freshwater environments for both ecosystem and human health. Many environmental abiotic and biotic variables can affect, singly and combined, the abundance and spread of AR determinants in freshwater ecosystems (in many cases, synergistic, additive, and antagonistic effects are detected), indicating that environmental AR monitoring programs need to be carefully adjusted to the particular casuistry of each freshwater ecosystem, as well as to the specific questions, interests, and resources of the corresponding program.

## Figures and Tables

**Table 1:** Strengths and limitations of most common current methods for studying antibiotic resistance in aquatic systems.

Method	Strengths	Limitations
Culture-based methods	Low technical and infrastructure requirements High sensitivity, detection of resistant bacteria at low abundances Direct measure of phenotypic resistance and physiological responses Direct link of resistance types to specific hosts Isolation of viable strains for further analysis	Limited to culturable organisms Bias towards fast-growing and dominant taxa under laboratory conditions Time-consuming and labour-intensive Low throughput compared to molecular approaches
qPCR technologies	Culture-independent Fast and accurate High sensitivity High specificity Requires small DNA amounts High comparability across samples and studies	Restriction to predetermined target genes Limited to established AR targets, novel or unknown genes not detected Primer and probe design requires expertise Expensive reagents Lack of information on gene expression Intracellular and extracellular DNA not distinguished Live and dead cells discrimination not possible Genetic context not determined
Targeted sequencing (amplicon-based metabarcoding)	Culture-independent Cost-effective compared to metagenomics High resolution of specific gene regions Useful for taxonomic profiling and targeted ARG surveillance	Limited to target regions Bioinformatics expertise required PCR bias may affect community representation
Whole genome sequencing	Comprehensive genomic information per isolate Identification of resistance mechanisms and mobile genetic elements High-resolution typing and epidemiological tracking	Limited to culturable organism, pure isolates required Time-consuming and labour-intensive Bioinformatics expertise required Reference databases required High sequencing costs
Shotgun metagenomics sequencing	Culture-independent Not limited to any pre-established set of genes or target regions Simultaneous characterisation of resistome and microbiome Expanded to latent AR targets, enables detection of novel ARGs Functional annotation PCR-free library preparations avoids amplification biases Genetic context and bacterial hosts elucidated via assembly-based approaches Data can be retrospectively analysed	High bioinformatics expertise, computational power and data storage required Time-consuming and labour-intensive Often dependent on a facility for sequencing analysis Live and dead cells discrimination not possible Reference databases required Lack of standardization in pipelines, databases and normalization High sequencing depth required for rare bacteria or genes High sequencing costs

## Data Availability

No new data were created or analyzed in this study. Data sharing is not applicable to this article.

## Supplementary Materials

The following supporting information can be downloaded at: https://www.mdpi.com/article/10.3390/antibiotics14080840/s1, Table S1: methodological details corresponding to Table 2; Table S2: methodological details corresponding to Table 4.

## Abbreviations

The following abbreviations are used in this manuscript:

3C	Chromosome Conformation Capture
AR	Antibiotic Resistance
ARB	Antibiotic-Resistant Bacteria
ARG	Antibiotic Resistance Gene
CARD	Comprehensive Antibiotic Resistance Database
DO	Dissolved Oxygen
ENA	European Nucleotide Archive
EpicPCR	Emulsion, Paired Isolation and Concatenation Polymerase Chain Reaction
FACS	Fluorescence-Activated Cell Sorting
FAIR	Findable, Accessible, Interoperable, and Reusable
FIB	Faecal Indicator Bacteria
FST	Faecal Source Tracking
HGT	Horizontal Gene Transfer
MAG	Metagenomic Assembled Genomes
MGE	Mobile Genetic Element
MRG	Metal Resistance Gene
MST	Microbial Source Tracking
NGS	Next-Generation Sequencing
PICT	Pollution-Induced Community Tolerance
qPCR	Real-time Quantitative PCR
ROS	Reactive Oxygen Species
SRA	Sequence Read Archive
WWTP	Wastewater Treatment Plant
